# Risk Categories for Discharge Planning Using AM-PAC "6-Clicks" Basic Mobility Scores in Non-Surgical Hospitalized Adults

**DOI:** 10.7759/cureus.69670

**Published:** 2024-09-18

**Authors:** Adele L Myszenski, George Divine, Jessica Gibson, Preethy Samuel, Michael Diffley, Anqi Wang, Aamir Siddiqui

**Affiliations:** 1 Rehabilitation, Henry Ford Health System, Detroit, USA; 2 Public Health Sciences, Henry Ford Health System, Detroit, USA; 3 Occupational Therapy, Wayne State University, Detroit, USA; 4 Plastic and Reconstructive Surgery, Henry Ford Health System, Detroit, USA; 5 Surgery, Henry Ford Health System, Detroit, USA

**Keywords:** am-pac 6-clicks, clinical decision support, effective hospital discharge planning, functional mobility scoring, patient-centered outcomes research, risk prediction

## Abstract

Background: Early discharge planning is important for safe, cost-effective, and timely hospital discharges. Patients with deconditioning are at risk for prolonged lengths of stay related to discharge needs. Functional mobility outcome measures are associated with discharge disposition. The purpose of this study is to examine the clinical usefulness of risk categories based on the Activity Measure for Post-Acute Care (AM-PAC) “6-clicks” Basic Mobility (6cBM) scores on predicting discharge destination.

Methods: A retrospective cohort study of 3739 adults admitted to general medical units at an urban, academic hospital between January 1, 2018 and February 29, 2020 who received at least two physical therapy visits and had an AM-PAC 6cBM recorded within 48 hours of admission and before discharge. The outcome variable was discharge destination dichotomized to post-acute care facilities (PACF); inpatient rehabilitation, skilled nursing facility, or subacute rehabilitation) or home (with or without home care services). The predictor variables were 6cBM near admission and discharge. Logistic regression was used to estimate the odds of being discharged to PACF compared to home, based on the Three-level risk categorization system: (a) low (6cBM score > 20), (b) moderate (6cBM score 15-19), or (c) high (6cBM score < 14) risk.

Results: Analysis indicated important differences between the three risk categories in both time periods. Based on 6cBM at admission, patients in the high-risk category were nine times more likely to be discharged to PACF than those in the low-risk category. At discharge, those in the high-risk category were 29 times more likely to go to PACF than those in the low-risk category. Other characteristics differentiating patients who went to PACF were sex (males), age (older) and longer hospitalization.

Conclusions: Predicting risk for discharge to a PACF using risk categories based on AM-PAC 6cBM can be useful for early discharge planning.

## Introduction

Discharge planning is a necessary component of acute care hospitalization, particularly as costs rise and pressures increase to reduce lengths of stay (LOS) and improve outcomes [1,2]. Nearly 40% of hospitalized patients require post-acute care (PAC) nursing or rehabilitation after hospital discharge with a disproportionate amount of payer spending on skilled nursing facilities (SNF) [3]. Individuals discharged to SNF have been reported to have increased lengths of stay and cost $1,800 more on average than those who were discharged home [4]. Impaired functional status as a result of hospital-acquired deconditioning (decreases in muscle strength and mass, cognitive and physical function) or worsening of baseline functional impairments may impact an individual’s ability to return to a home setting and may require acute care rehabilitation services [5-8].

Emerging evidence indicates that the functional mobility status of patients in acute care settings can predict the need for discharge to PAC facilities [9]. The Activity Measure for Post-Acute Care (AM-PAC) 6-clicks Basic Mobility (6cBM) short form is one such tool used to predict discharge disposition [8,10-16].

Healthcare professionals, patients, and family members need high-quality, timely data and resources to understand medical decision-making, prognosis, and outcomes. Those receiving the information need enough data to act without being overwhelmed by information overload, because singular scores may not reflect multi-factorial aspects of discharge planning. Past studies using singular cut-points to associate scores with discharge dispositions have indicated that lower mobility scores are associated with a higher likelihood of discharge to post-acute care facilities (PACF) [8,10,16-18]. Stratifying mobility scores into risk categories can be more clinically relevant and useful. Many risk assessment tools are used in health care to predict pressure ulcers, venous thromboembolism, and surgical discharge needs [19-23].

The purpose of this study was to examine the predictive utility of AM-PAC 6cBM scores by physical therapy (PT) professionals at the first and last visit, using risk categories defined as (a) low (AM-PAC I6cBM score >20), (b) moderate (AM-PAC 6cBM score 15-19), or (c) high (AM-PAC 6cBM score <14) risk. It was hypothesized that the high-risk category would be more likely to go to PACF than those in the low-risk category.

## Materials and methods

Study population and design

This study used a retrospective cohort study design with data extracted from electronic health records of an urban tertiary care hospital. The study procedures were approved by the Henry Ford Health Institutional Review Board (14107-01) with waived consent to access records. Data was collected for all patients aged 18 years or older admitted to a general medical unit between January 1, 2018, and February 29, 2020. Exclusion criteria included leaving against medical advice, expiring in the hospital, entered hospice, or transferred to another acute care hospital. To reduce the confounder of severity, patients were also excluded if the length of hospital stay was less than one day or greater than 45 days or had diagnoses indicating surgical procedures.

Instrument

Mobility was measured by physical therapists (PTs) or PT assistants at every visit using the AM-PAC 6cBM scale. The 6cBM has been described as a reliable and valid tool, easy to use by various healthcare professionals [21-24]. A four-point scale is used to assess the amount of assistance (1 = total assistance, 2 = lot of assistance, 3 = little assistance, and 4 = no assistance) needed to complete six mobility tasks (rolling, bed mobility, transfers, standing, walking and stair climbing). Raw scores range from 6 to 24, with higher scores indicating better mobility. Scores are collected in the EHR as mandatory documentation for all visits. All PTs and PT assistants received one-time didactic training on the tool using the AM-PAC Instruction Manual version 4.0 in November 2017 or at the time of hire. Annual audits for accuracy and compliance are completed [25]. 

Using consensus-building methodology, terminology was refined to develop a three-level risk scale*. *To develop a risk assessment tool that would be clinical relevant to our setting, a multidisciplinary workgroup was established, consisting of physical therapy, occupational therapy, nursing, case management, a physician advisor and a data analyst programmer. The workgroup met over the course of four months and reviewed evidence, including five single-score predictive utility studies and a PowerPoint presentation delivered by leaders at the Cleveland Clinic. An exploratory analysis of single score and discharge destination was conducted and using consensus-building methodology, a 3-level risk scale was developed [8,10,16-18]. Scores less than or equal to 14 were considered high-risk for requiring PACF at discharge based on tool descriptions and clinical assumptions for patients requiring total or a lot of assistance in all areas of mobility [25]. Scores ranging from 15 to 19 were categorized at moderate risk for discharge to PACF as patients require a little to a lot of physical assistance. Scores greater than or equal to 20 were considered low risk, as patients in this group require little to no assistance with mobility tasks. These designations were based on the 6cBM operational definitions, clinical relevance, and ease of communication for all healthcare stakeholders [26].

Variables of interest

The outcome variable for this study was discharge destination that was dichotomized to either PACF (inpatient rehabilitation facility, skilled nursing facility, or subacute rehabilitation facility) or home (home with or without home care services). The predictor variables were patients’ 6cBM mobility scores at admission and discharge. First and last therapist AM-PAC 6cBM scores were utilized. For patients who received therapy, these scores were often within 48 hours of hospital admission and 48 hours of hospital discharge. The sociodemographic variables were patient (age, sex, and race), and length of stay (hospital and intensive care unit (ICU)) characteristics.

Statistical analysis

Data extracted from electronic health records was analyzed, and risk was presented using nonparametric tests (Wilcoxon rank sum or Pearson's Chi-square) to compare patient characteristics based on discharge destination. Univariate logistic regression was used to estimate the odds ratio between each two-level pair of risk levels at admission and at discharge. Odds ratios, 95% Confidence Interval (CI) and area under the curve were also determined. The SAS software (SAS Institute, Inc., Cary, NC) was used for analyses. Nonparametric tests (Wilcoxon rank sum or Pearson's Chi-square) were used to compare patient characteristics based on discharge destination. Univariate logistic regression was used to estimate the odds ratio between each two-level pair of risk levels at admission and at discharge.

## Results

Sample description

The total number of patients meeting inclusion criteria was 6849. Patients who did not have a mobility evaluation within 48 hours of admission (n = 3067) and at discharge (n = 43) were also excluded. The final sample of interest for this study was 3739 patients with non-surgical diagnoses admitted to general medical units and had 6cBM scores at 48 hours from admission and at discharge. The demographic and clinical characteristics of the population are provided in Table 1. The mean age of the sample was 65.2 years (standard deviation (SD) = 14.8). Females (51%) and Black race (63%) comprised the majority of the sample. The “other race” category included American Indian/Aleutian, Asian, Hispanic, Middle Eastern, Multi-racial, Pacific Islander/Hawaiian, and other unidentified ethnic groups. The average length of hospital stay was 7.8 days (SD = 5.4), and the average length of ICU stay for those who needed ICU care was 1.9 days (SD = 3.1).

**Table 1:** Demographic and medical status by discharge destination ICU, intensive care unit; PACF, post-acute care facilities; SD, standard deviation. ^1^For all patients, column percentages are shown; ^2^For PACF and Home, row percentages are shown; ​​​​​​​^3^By Wilcoxon rank sum test or by Pearson’s chi-square test. Note: All patients have a diagnosis-related group (DRG) in a non-surgical medical group and were Admitted or Discharged from medical floors only.

		Patients by Discharge Destination		
Variable	All Patients N = 3739 Mean ± SD; n (%)^1^	PACF N = 1694 Mean ± SD; n (%)^2^	Home N = 2045 Mean ± SD; n (%)^2^	p-value^3^
Age, years	65.2 ± 14.8	68.8 ± 13.7	62.2 ± 15.1	<0.001
Sex, Female	1910 (51.1%)	829 (43.4%)	1081 (56.6%)	0.017
Sex, Male	1829 (48.9%)	865 (47.3%)	964 (52.7%)	
Black race	2259 (63.1%)	1020 (45.2%)	1239 (54.9%)	<0.001
White race	1129 (31.5%)	545 (48.3%)	584 (51.7%)	
Other/Unknown race	191 (5.3%)	129 (36.8%)	222 (63.3%)	
Length of hospital stay (days)	7.8 ± 5.4	9.3 ± 6.0	6.6 ± 4.5	<0.001
Length of ICU stay (days)	1.9 ± 3.1	1.8 ± 3.4	1.9 ± 2.8	<0.001

Of the 3739 patients in the sample of interest, 1694 (45.3%) were discharged to a PACF. Those discharged to a PACF tended to be older (mean 68.8 years, SD = 13.7) than those who went home (mean 62.2 years, SD = 15.1). Of the 3739 patients in the study sample, 829 (22.2%) females and 865 (28.9%) males were discharged to PACF. Black race (n = 1020, 28.5), White (n = 545, 15.2%), and other ethnic groups (n = 53, 1.4%) were among the 3579 patients (race was not reported for 160 patients) who were discharged to PACF. Patients who were discharged to PACF had longer hospital stays (9.3 days) than those who went home (6.6 days); however, the average ICU stay was similar for both categories.

Description of risk categories based on admission mobility scores

Table 2 summarizes the proportions of patients discharged to PACF compared to the total number of patients in each mobility-based risk category at admission and discharge. The mobility scores at admission indicated that 521 of the 3739 (13.9%) patients in the sample were categorized to be at low risk for discharge to a PACF, followed by 1797 (48.1%) at moderate risk and 1421 (38%) at high risk. Based on admission mobility scores, it appears that the probability of discharge to PACF was higher for patients in the high-risk category (66.1%) than for patients in the moderate (37.0%) and (17.3%) low-risk categories. 

**Table 2:** Comparison of PACF discharges by mobility-based risk categories at admission and discharge PACF, post-acute care facilities. n1 = number of patients discharged to PACF; n2 = number of patients who scored within the risk categorization. ^1^Admission mobility score at first physical therapy visit (within 48 hours of admission); ^2^Discharge mobility score recorded at last physical therapy visit prior to discharge.

Basis for Risk Category	Low Risk (>20)	Moderate Risk (15-19)	High Risk (<14)	Chi-square test p-value
	n1/n2 (%)	n1/n2 (%)	n1/n2 (%)	
Admission mobility score^1^	90/521 (17.3%)	665/1797 (37.0%)	939/1421 (66.1%)	<0.001
Discharge mobility score^2 ^	116/1071 (10.8%)	787/1652 (47.6%)	791/1016 (77.9%)	<0.001

Likelihood of discharge to PACF based on admission mobility scores 

Table 3 indicates that patients in the high-risk category, based on admission mobility scores, were 9.33 times more likely than those in the low-risk category and 3.3 times more likely than those in the moderate-risk category to be discharged to a PACF. Those in the moderate-risk category at admission were 2.8 times more likely to be discharged to a PACF than those in the low-risk category.

**Table 3:** Odds ratios (unadjusted) comparing three risk categories at admission and discharge by discharge destination to home

	Odds Ratio	95% Confidence Limits	
Admission Timepoint, High vs Low	9.3	7.2	12.0
Admission Timepoint, Moderate vs Low	2.8	2.2	3.6
Admission Timepoint, High vs Moderate	3.3	2.9	3.8
Discharge Timepoint, High vs Low	28.9	22.7	36.9
Discharge Timepoint, Moderate vs Low	7.5	6.0	9.3
Discharge Timepoint, High vs Moderate	3.9	3.2	4.6

Description of risk categories based on discharge mobility scores

Table 2 indicates that when patients in this study were assessed closer to discharge, 28.6% (1071 out of 3739) were categorized to be at low risk, 44.2% (1652 out of 3739) at moderate risk, and 27.1% (1016 out of 3739) of high risk to be discharged to a PACF. About 10.8% of patients in the low-risk category based on discharge mobility score were discharged to a PACF, followed by 48% of those in the moderate-risk category and 78% of those in the high-risk category were discharged to a PACF.

Likelihood of discharge to PACF based on discharge mobility scores

Table 3 indicates that patients in the high-risk category, based on discharge mobility scores were 28.9 times more likely than those in the low-risk category and 7.5 times more likely than those in the moderate-risk category to be discharged to a PACF. Those in the moderate-risk category at admission were 3.9 times more likely to be discharged to a PACF than those in the low-risk category.

Comparison of 19-level scores versus three-levels

**Figure 1:**
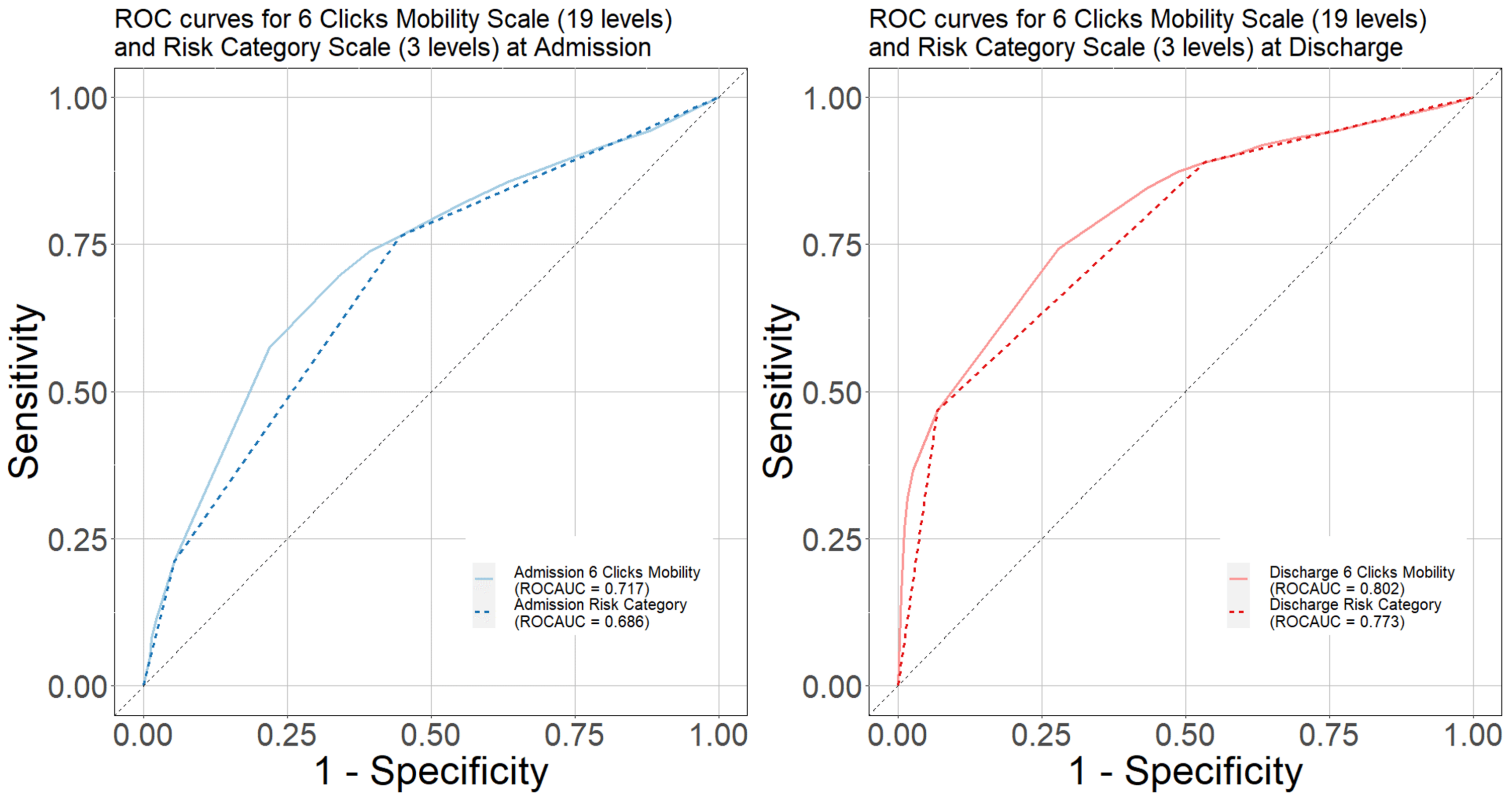
Receiver operating characteristics and areas under the curve for 6-Clicks – 19 level raw scores and 3 level risk category Abbreviation: ROC, receiver operating characteristic.

To assess the predictive ability of the three-category risk level, compared to the 19-level 6-clicks mobility scale, Figure 1 shows their respective receiver operating characteristic curves plotted together, both near admission (left panel) and near discharge (right panel). As expected, the 19-level scale has a greater receiver operating characteristic area under the curve than the three-level grouping at each time point, 0.717 vs 0.686 and 0.802 vs 0.773, respectively. However, the differences are not particularly large.

## Discussion

This study developed and examined the utility of distinct three-category categorization (low, moderate, and high) of the AM-PAC 6cBM scale for patients admitted to general medicine units in a large tertiary hospital and received at least two physical therapy visits during their hospital stay. Single score cut-points for the predictive utility of the AM-PAC 6cBM have been examined previously in the literature, which highlights the acceptance of measures of functional mobility as predictive and likely clinically useful in identifying risk [6,8,10-12,15,17,26-28]. Herbold et al. sought to establish cutoff scores for various diagnostic groups and found a single score was sufficient, but not all groups conformed to the single cutoff score, in this case, AM-PAC t-score of 42.88 for 6cBM (conversion to raw scores between 17 and 18) [26]. This study represents the evolution of this by providing the necessary nuance and risk categorization. The distinct categories can be used to stratify the probability of discharging to a post-acute care facility (PACF), including inpatient rehabilitation, subacute rehabilitation, and skilled nursing facilities. Patients in the low-risk category (6cBM >20) were 9 to 29 times less likely to be discharged to a PACF than those in the high-risk category (6cBM <14).

Hospital-associated deconditioning can impact all patients to some degree, and the goal was to better understand the phenomena and quantify them through stratification of the 6cBM score [7]. As previously mentioned, the 6cBM is a validated and accepted method of representing a patient’s physical abilities [21-23]. The assessment of physical mobility function is information not only for discharge planning but also for a predictor of morbidity and mortality. Functional status at time of discharge has been shown to be predictive of out-of-hospital mortality with low functional status associated with an almost seven-fold increase in 90-day mortality when compared to high functioning critically ill patients [29]. Given the significance of mobility and functionality, it is vital that patients are discharged to a facility that can provide the appropriate level of care if indicated. For patients that require PACF placement, the process can be lengthy and has the very real potential of increasing hospital stay and thereby exposing the patient to more nosocomial risks; the value of an accurate predictive disposition model, which can use an early admission evaluation to identify patients who will likely need post-discharge rehabilitation, cannot be understated.

This study’s results correlate with previous large center studies of the predictive utility of the AM-PAC instruments. Warren et al. studied AMPAC predictive ability with respect to discharge destination and found area under the curve values of 0.80 (95% CI, 0.80-0.81) and 0.81 (95% CI, 0.80-0.82) for Basic Mobility and Daily Activity, respectively [8]. They used a cut point of 16 for Basic Mobility and 19 for Daily Activity. They found that patients with a Basic Mobility score below the threshold were 7.8 (95% CI, 6.83-8.91) times more likely to be discharged to a skilled nursing facility than home, and those with a Daily Activity score below threshold were 8.87 (95% CI, 7.9-9.95) times more likely to be discharged to a skilled nursing facility. Odds ratios for discharge to inpatient rehabilitation facility were 7.54 (95% CI, 6.28-8.91) and 11.44 (95% CI, 9.68-13.51) for Basic Mobility and Daily Activity, respectively [8]. Myszenski et al. indicated that patients with an AMPAC score of 16 or higher need little to no assistance to perform basic mobility and daily activities, are more likely to return home at discharge than those with scores of 15 or lower [27]. Gore et al. investigated the predictability of the AM-PAC tools for patients hospitalized with chronic obstructive pulmonary disease and also found AM-PAC tools to be strong predictors of discharge disposition as compared to age, sex, or insurance type. They concluded that 6cBM scores at discharge were able to detect with nearly 73% accuracy if the patient would be discharged home independently [14]. 

Timing of score predictability was also examined. Discharge planning begins at the time of acute care admission. Patients, caregivers, and care professionals often must consider the options and impact of care requirements on hospital care and recovery. The earlier relevant information is assessable, the more actionable it is. We looked at 6cBM performed close to admission and close to discharge. Not surprisingly, high-risk 6cBM at the last timepoint was most predictive of discharge to PACF (77.9%) (Table 2). This timepoint is the closest to actual hospital discharge and because of its proximity to discharge, it is also the least actionable. The first timepoint categories may be more useful for predicting need for post-acute care as opposed to likelihood of discharge to home. For the first timepoint, the high-risk population to PACF was 66.1% (Table 2). We evaluated the impact of timing on the mobility score. Not surprisingly, assignment to the high-risk 6cBM group at the last timepoint (before discharge) was most predictive of discharge to PACF (78%) (Table 2). This timepoint is the closest to actual hospital discharge and therefore the least actionable from a planning standpoint. At the other end, assignment to the high-risk cohort at the first timepoint (after admission) resulted in 66% predictive value for disposition to PACF (Table 2). This is still very helpful. Pfoh et al. also studied initial AM-PAC Basic Mobility scores taken within 48 hours of hospital admission and concluded that initial scores had moderate ability to predict discharge destination, with an odds ratio of 0.78 [15]. This early information can be used to allocate resources, educate the patient and family, and begin the logistical and financial planning that can delay and complicate the discharge process from the acute care setting.

In the second time point, there was a between categories shift, with an increase of scores recorded in the low-risk category and a decrease in the moderate and high risk categories (Table 2). This indicates a positive change in functional status based solely on the AM-PAC basic mobility score. The improvement in overall AM-PAC score distributions, with more patients in the higher score/lower risk group, suggests improved functional status during hospitalization, better functional status near discharge, and the potential impact of physical therapy acutely. Future research is underway to investigate if a more focused resource allocation can improve patient mobility and functional abilities during the hospital stay.

Risk stratification using AM-PAC 6cBM offers an opportunity to use data in a clear concise manner and to use clinical decision support to reduce information overload in the healthcare environment. The goal should be to give information to the care team that allows them to make informed decisions. Understanding the impact of patient deconditioning is a step in that direction. Our results confirm that the first timepoint measurement provides a reasonable proxy for mobility and ability at the time of discharge. Further work is needed to apply this paradigm in a prospective model.

Limitations

Although only a single institution’s experience, the large number of patients and the hundreds of care providers used for patient assessments should imply broad applicability. Further limitations include the retrospective nature of the study design, lack of covariates such as patient’s origin (home or PACF) and selection bias. Analysis was only completed for patients who received PT at least two times, and AM-PAC scores were completed only by PTs, thereby limiting the generalizability to all patients or those with scores entered by other healthcare professionals. The data collected was not primarily collected for the purposes of research and was vulnerable to misclassifications and coding errors, however, errors were mitigated with peer chart reviews and random audits. The percentages and odds ratios are unadjusted estimates, however the very strong (statistically significant) associations would be unlikely to lose significant if adjustment were to be made. Actual discharge destination can be multi-factorial, including social determinants of health, individual preferences, and financial constraints. The timing of scoring was not standardized and could have influenced results.

## Conclusions

Our study shows that AM-PAC 6cBM scores can be used near admission and near discharge to identify hospitalized medicine patients more or less likely to require post-acute discharge rehabilitative care at hospital discharge. Risk categorization into three groups: (a) low (AM-PAC 6cBM score >20), (b) moderate (AM-PAC 6cBM score 15-19), or (c) high (AM-PAC 6cBM score <14) displayed predictive utility similar to single scores. Patients with lower mobility scores are more likely to be discharged to a PACF than those with higher mobility scores. Earlier identification of risk for PACF, based on a range of AM-PAC 6cBM scores, maybe a useful tool for discharge disposition planning and resource allocation.

## References

[1] Gonçalves-Bradley DC, Lannin NA, Clemson L, Cameron ID, Shepperd S (2022). Discharge planning from hospital. Cochrane Database Syst Rev.

[2] Shepperd S, Lannin NA, Clemson LM, McCluskey A, Cameron ID, Barras SL (2013). Discharge planning from hospital to home. Cochrane Database Syst Rev.

[3] Hajduk AM, Murphy TE, Geda ME (2019). Association between mobility measured during hospitalization and functional outcomes in older adults with acute myocardial infarction in the SILVER-AMI study. JAMA Intern Med.

[4] Hoyer EH, Needham DM, Atanelov L, Knox B, Friedman M, Brotman DJ (2014). Association of impaired functional status at hospital discharge and subsequent rehospitalization. J Hosp Med.

[5] Tian W (2016). Statistical Brief #205 An All-Payer View of Hospital Discharge to Postacute Care, 2013. HCUP Nationwide Inpatient Sample (NIS).

[6] Werner RM, Coe NB, Qi M, Konetzka RT (2019). Patient outcomes after hospital discharge to home with home health care vs to a skilled nursing facility. JAMA Intern Med.

[7] Junek ML, Jones A, Heckman G, Demers C, Griffith LE, Costa AP (2022). The predictive utility of functional status at discharge: a population-level cohort analysis. BMC Geriatr.

[8] Warren M, Knecht J, Verheijde J, Tompkins J (2021). Association of AM-PAC "6-Clicks" basic mobility and daily activity scores with discharge destination. Phys Ther.

[9] Tevald MA, Clancy MJ, Butler K, Drollinger M, Adler J, Malone D (2021). Activity measure for post-acute care "6-Clicks" for the prediction of short-term clinical outcomes in individuals hospitalized with COVID-19: a retrospective cohort study. Arch Phys Med Rehabil.

[10] Jette DU, Stilphen M, Ranganathan VK, Passek SD, Frost FS, Jette AM (2014). AM-PAC "6-Clicks" functional assessment scores predict acute care hospital discharge destination. Phys Ther.

[11] Covert S, Johnson JK, Stilphen M, Passek S, Thompson NR, Katzan I (2020). Use of the activity measure for post-acute care "6 Clicks" basic mobility inpatient short form and National Institutes of Health Stroke Scale to predict hospital discharge disposition after stroke. Phys Ther.

[12] Casertano LO, Bassile CC, Pfeffer JS, Morrone TM, Stein J, Willey JZ, Rao AK (2022). Utility of the AM-PAC "6 Clicks" basic mobility and daily activity short forms to determine discharge destination in an acute stroke population. Am J Occup Ther.

[13] Blackwood J, Fernandez N (2018). AM-PAC 6-clicks scores predict hospital discharge destination in older adults with cardiovascular disease. Innov Aging.

[14] Gore S, Blackwood J, Emily H, Natalia F (2023). Determinants of acute care discharge in adults with chronic obstructive pulmonary disease. Physiother Theory Pract.

[15] Pfoh ER, Hamilton A, Hu B, Stilphen M, Rothberg MB (2020). The six-clicks mobility measure: a useful tool for predicting discharge disposition. Arch Phys Med Rehabil.

[16] LeBrun DG, Nguyen JT, Fisher C (2023). The risk assessment and prediction tool (RAPT) score predicts discharge destination, length of stay, and postoperative mobility after total joint arthroplasty. J Arthroplasty.

[17] Tuohy S, Schwartz-Dillard J, McInerney D, Nguyen J, Edwards D (2024). RAPT and AM-PAC "6-Clicks": do they correlate on predicting discharge destination after total joint arthroplasty?. HSS J.

[18] Piazza M, Sharma N, Osiemo B (2019). Initial assessment of the risk assessment and prediction tool in a heterogeneous neurosurgical patient population. Neurosurgery.

[19] Moore ZE, Patton D (2019). Risk assessment tools for the prevention of pressure ulcers. Cochrane Database Syst Rev.

[20] Pandor A, Tonkins M, Goodacre S (2021). Risk assessment models for venous thromboembolism in hospitalised adult patients: a systematic review. BMJ Open.

[21] Jette DU, Stilphen M, Ranganathan VK, Passek S, Frost FS, Jette AM (2015). Interrater reliability of AM-PAC "6-Clicks" basic mobility and daily activity short forms. Phys Ther.

[22] Jette DU, Stilphen M, Ranganathan VK, Passek SD, Frost FS, Jette AM (2014). Validity of the AM-PAC "6-Clicks" inpatient daily activity and basic mobility short forms. Phys Ther.

[23] Johnson JK, Lapin B, Bethoux F, Skolaris A, Katzan I, Stilphen M (2022). Patient versus clinician proxy reliability of the AM-PAC "6-Clicks" basic mobility and daily activity short forms. Phys Ther.

[24] Hoyer EH, Young DL, Klein LM (2018). Toward a common language for measuring patient mobility in the hospital: reliability and construct validity of interprofessional mobility measures. Phys Ther.

[25] Jette A, Haley SM, Coster W, Pengsheng N (2020). AM-PAC Short Forms Manual 4.0.

[26] Herbold J, Rajaraman D, Taylor S, Agayby K, Babyar S (2022). Activity measure for post-acute care "6-Clicks" basic mobility scores predict discharge destination after acute care hospitalization in select patient groups: a retrospective, observational study. Arch Rehabil Res Clin Transl.

[27] Myszenski A, Zhou Y, Abbas FT, Siddiqui A (2022). The predictive validity of functional outcome measures with discharge destination for hospitalized medical patients. Arch Rehabil Res Clin Transl.

[28] Young DL, Colantuoni E, Friedman LA (2020). Prediction of disposition within 48 hours of hospital admission using patient mobility scores. J Hosp Med.

[29] Rydingsward JE, Horkan CM, Mogensen KM, Quraishi SA, Amrein K, Christopher KB (2016). Functional status in ICU survivors and out of hospital outcomes: a cohort study. Crit Care Med.

